# Prognostic value of the New York Heart Association classification for cardiovascular events and mortality in Chagas cardiomyopathy: a systematic review and meta-analysis with GRADE recommendations

**DOI:** 10.1590/0037-8682-0104-2026

**Published:** 2026-08-03

**Authors:** Whesley Tanor Silva, Henrique Silveira Costa, Hytalo de Jesus Silva, Matheus Ribeiro Ávila, Lucas Frois Fernandes Oliveira, Juliana Pereira Silva, Sandylere Moreira Baia Gomes, Alejandro Marcel Hasslocher-Moreno, Pedro Henrique Scheidt Figueiredo, Mauro Felippe Felix Mediano

**Affiliations:** 1 Fundação Oswaldo Cruz, Instituto Nacional de Infectologia Evandro Chagas, Rio de Janeiro, RJ, Brasil.; 2 Universidade Federal dos Vales do Jequitinhonha e Mucuri, Departamento de Fisioterapia, Diamantina, MG, Brasil.; 3 Universidade Federal de Minas Gerais, Faculdade de Medicina, Belo Horizonte, MG, Brasil.; 4 Fundação Oswaldo Cruz, Instituto Oswaldo Cruz, Rio de Janeiro, RJ, Brasil.

**Keywords:** Chagas cardiomyopathy, Chagas disease, NYHA, Prognoses, Prognostic factor, Systematic review

## Abstract

Chagas cardiomyopathy is associated with a higher risk of severe cardiovascular events and increased mortality. Among the factors associated with Chagas cardiomyopathy, impairment in functional capacity-commonly assessed using the New York Heart Association (NYHA) functional classification-is notable and may contribute to risk stratification. In this review, we assessed the prognostic value of the NYHA classification on cardiovascular events and mortality in patients with Chagas cardiomyopathy. The search was performed across EMBASE, LILACS, MEDLINE, and Web of Science databases. Prospective and retrospective cohort studies in which the prognostic value of the NYHA classification was assessed were eligible. Risk of bias was assessed using the Quality in Prognostic Studies tool. Evidence was classified using the adapted GRADE system. Eighteen studies were included in the systematic review. The meta-analysis results indicate that the NYHA classification is an important prognostic factor for mortality. Patients with NYHA class III and IV showed a higher risk of death than those with class I and II (hazard ratio, 2.63; 95% confidence interval: 2.00-3.45; moderate GRADE evidence). Patients with NYHA class IV had a higher risk of death than those with class I, II, and III (hazard ratio, 2.80; 95% confidence interval: 1.06-7.43; low GRADE evidence). Very low evidence suggests that the NYHA classification does not predict heart transplantation or stroke, but may predict cardiac pacemaker implantation. Overall, the NYHA classification is a key prognostic indicator of mortality in Chagas cardiomyopathy, although its ability to predict stroke, heart transplantation, and pacemaker implantation is inconsistent.

## INTRODUCTION

Chagas disease, a neglected tropical disease^1^ ^,^ ^2^, an important public health problem in Latin America^1^ and, more recently, in non-endemic areas such as the United States and some European countries^3^. Approximately 70 million people are at risk of infection, with an incidence of 30,000 cases and approximately 12,000 deaths occurring annually^4^. The infection begins when an individual is exposed to *Trypanosoma cruzi*, and advances to the acute disease phase, which is usually characterized by nonspecific clinical manifestations^5^. The infection later progresses to the chronic phase, which can be divided into indeterminate (i.e., without evidence of organ involvement), cardiac (i.e., with specific heart abnormalities), digestive (i.e., with specific digestive abnormalities), or mixed (i.e., heart and digestive abnormalities simultaneously)^6^ forms.

About 30% to 40% of infected individuals present with the cardiac form of Chagas disease, also known as Chagas cardiomyopathy (ChC); this is the most common and severe clinical form^7^. Patients with ChC may have a worse prognosis than those with cardiomyopathies of other etiologies^8^. This worse prognosis can be attributed to the specific pathophysiological characteristics of Chagas disease-such as chronic cardiac inflammatory processes, dysautonomia, and cardiac tissue remodeling^9^-and to social determinants frequently associated with the disease, including limited access to specialized healthcare, low levels of education, and low income^9^ ^,^ ^10^. Together, these biological and social factors may contribute to poor clinical outcomes in this population, including the need for pacemaker implantation or heart transplantation, and the occurrence of cardioembolic events and sudden death^11^ ^,^ ^12^.

During the clinical course of ChC, patients experience a significant reduction in their capacity to exercise^13^. This reduction in functional capacity often leads to lower levels of activity, especially when symptoms such as angina and dyspnea are present^11^. This may cause a cycle to develop between inactivity and symptoms of ChC, further reducing functional capacity. Consequently, besides information on the current functional status, symptom intensity during exertion can be an additional important prognostic factor for progression of the condition^14^. Among the tools used to assess functional symptoms, the New York Heart Association (NYHA) functional classification is the most frequently cited in the literature. This classification is a low-cost, easy-to-use tool. The NYHA tool is used to classify symptoms into four categories based on the severity of the patient’s symptoms and their ability to perform physical activities; these categories range from class I (*no symptoms*) to class IV (*severe symptoms even at rest*)^15^. The NYHA functional class is considered a prognostic factor for death and hospitalization caused by other heart diseases. In ChC, it is known to be highly associated with markers of severity, such as reduced left ventricular ejection fraction^16^ and peak oxygen uptake. Of the predictors of death identified in a cohort of 424 patients with ChC, NYHA functional classes III and IV were found to be the strongest^17^. By contrast, other studies^18^ ^-^ ^20^ have reported that the NYHA class does not identify patients with a worse prognosis. Therefore, the prognostic value of the NYHA class in predicting cardiovascular events and mortality in patients with ChC is uncertain^21^ ^,^ ^22^.

Prognostic factors are variables associated with the risk of a future health outcome, indicating either a better or worse prognosis^23^. Identifying these variables is important for clinical decision-making, facilitating the adoption of tailored interventions and providing research targets for new treatments in randomized trials^24^. Given the importance of functional assessment and the uncertainty regarding the prognostic value of the NYHA class in patients with ChC, we aimed to evaluate the prognostic value of the NYHA class for cardiovascular events and mortality in this population in this systematic review.

## METHODS

This systematic review followed the methods proposed by the Cochrane Prognosis Methods Group^25^ ^,^ ^26^ and is reported in accordance with the Preferred Reporting Items for Systematic reviews and Meta-Analyses (PRISMA) Statement^27^. The protocol was registered prospectively in the Open Science Framework (registration number 10.17605/OSF.IO/F578).

### Searches and inclusion criteria

The Literatura Latino-Americana e do Caribe em Ciências da Saúde (LILACS), Medical Literature Analysis and Retrieval System Online (MEDLINE), Excerpta Medica Database (EMBASE), and Web of Science databases were searched without language or date restrictions. The search terms were related to “Chagas cardiomyopathy” and “prognostic study.” Database-specific search strategies were developed for each source, considering its controlled vocabulary, syntax, indexing structure, and language of indexed terms. For MEDLINE, searched using the Ovid platform, we used the prognostic-factor search strategy recommended by the Cochrane Prognosis Methods Group (sensitivity, 0.98; specificity, 0.96)^28^. In addition to the main MEDLINE search, Epub Ahead of Print, In-Process, In-Data-Review and Other Non-Indexed Citations, Daily and Versions, and Biological Abstracts were selected as supplementary search sources to improve the identification of grey literature, and non-indexed and incompletely indexed records. To complement this strategy, we manually screened the reference lists of relevant systematic reviews to identify potentially eligible studies that were not retrieved through the electronic searches. The complete search strategies for each database, including terms, combinations, filters, limits, and update strategies, are provided in the Supplementary Material in accordance with the PRISMA-S, the extension to the PRISMA Statement for Reporting Literature Searches in Systematic Reviews.

After the searches were conducted, the references retrieved were exported to the Rayyan systematic review website (https://www.rayyan.ai/) to select studies based on defined eligibility criteria. Two independent reviewers (WTS and HJS) screened titles and abstracts, and then assessed potential full texts. Between-reviewer discrepancies were resolved by a third reviewer (HSC).

Prospective and retrospective cohort studies in which the prognostic value of the NYHA class on cardiovascular events and mortality were assessed were eligible, with no restrictions placed on date or language. Studies were eligible if they included individuals of both sexes, regardless of age, from any health care setting. The criteria for the population of interest were that Chagas disease was confirmed through positive serology for *T. cruzi* using at least two different serological tests, and that the electrocardiographic findings were compatible with Chagas cardiomyopathy^29^. For studies published before these criteria were adopted in 2005, diagnostic definitions accepted at the time of publication were considered when they were explicitly reported^30^. The index prognostic factor was higher NYHA functional class, defined as classifications representing greater symptom severity. NYHA classes representing less severity were used as the comparator and or reference group whenever possible. Therefore, effect estimates were interpreted as the risk of adverse outcomes among patients with more severe NYHA classes compared with those with less severe NYHA classes. Further details are provided in Table 1. Due to difficulties in obtaining data on individual participants, studies were grouped by their original NYHA classification to ensure homogeneity. The outcomes considered were adverse cardiovascular events (i.e., implantation of implantable cardiac electronic devices, heart transplantation, and stroke), and mortality. The formulation of the research question in the patient population, exposure (index prognostic factor), comparator, outcome, timing, and setting format is presented in Table 1.

**TABLE 1:** Patient population, prognostic factor (exposure), comparator, and outcome review questions.

Population	Individuals with Chagas cardiomyopathy.
Prognostic factor (exposure)	NYHA classifications representing greater symptom severity.
Comparator	Lower-severity NYHA classifications used as the reference/comparator group (e.g., NYHA class I-II as reference for NYHA class III-IV).
Outcomes	Mortality (primary outcome); cardiovascular events (secondary outcomes). The cardiovascular events considered were implantation of implantable cardiac electronic devices (e.g., pacemakers and implantable cardioverter-defibrillators), heart transplantation, and stroke

NYHA: New York Heart Association.

### Data extraction

Data extraction was performed according to the Critical Appraisal and Data Extraction for Systematic Reviews of Prediction Modelling Studies checklist. The variables for which data were collected included data origin, participants, cardiovascular outcomes, NYHA class, and sample size. Data extraction was conducted independently by two authors, and any discrepancies were resolved through discussion and, when necessary, adjudication by a third reviewer.

### Missing data

When data were missing, we contacted the original investigators to request the information. The authors were contacted three times, with a 1-week interval. When necessary, we calculated or estimated effect sizes from any reported data, for example, from 2 × 2 frequency tables, graphs, and figures, such as Kaplan-Meier curves; we used indirect estimation measures, as recommended by Parmar et al^31^. and Tierney et al^32^. If missing data could not be obtained through any of these approaches, the study was included in the systematic review but not in the meta-analysis.

### Risk of bias assessment

Two independent reviewers (WTS and HJS) assessed the risk of bias of the included studies using the Quality in Prognostic Studies (QUIPS) tool^33^. Briefly, the tool contains six domains: (i) study participation, (ii) study attrition, (iii) prognostic factor measurement, (iv) outcome measurement, (v) study confounding, and (vi) statistical analysis and reporting. Each domain was rated as having a high, moderate, or low risk of bias. Discrepancies were resolved by a third reviewer (HSC).

### Data analysis

When possible, meta-analyses were conducted using DerSimonian and Laird’s random-effects model because it was the most appropriate to use when heterogeneity among the studies was expected^34^. For the meta-analyses, we included only adjusted hazard ratios derived from the final multivariable models reported in each study. Studies reporting only crude or unadjusted estimates were retained in the descriptive synthesis, but excluded from the meta-analyses. Heterogeneity was assessed using the I² statistic, and Cochran’s Q test was used to assess the significance of the heterogeneity^35^. The results are presented as a forest plot. Details on the statistical analysis and scripts are summarized in the Supplementary Material. All analyses were performed using R 4.4.3 software.

Two independent reviewers (WTS and HJS) assessed the certainty of the current evidence using the adapted GRADE scale for prognostic studies^36^. Between-reviewer discrepancies were resolved by a third reviewer (HSC). According to the four-level GRADE system, evidence can range from high to very low quality, with low levels indicating that future high-quality trials are likely to change the estimated effects. The criteria applied in the GRADE assessment are provided in the Supplementary Material.

## RESULTS

**FIGURE 1:**
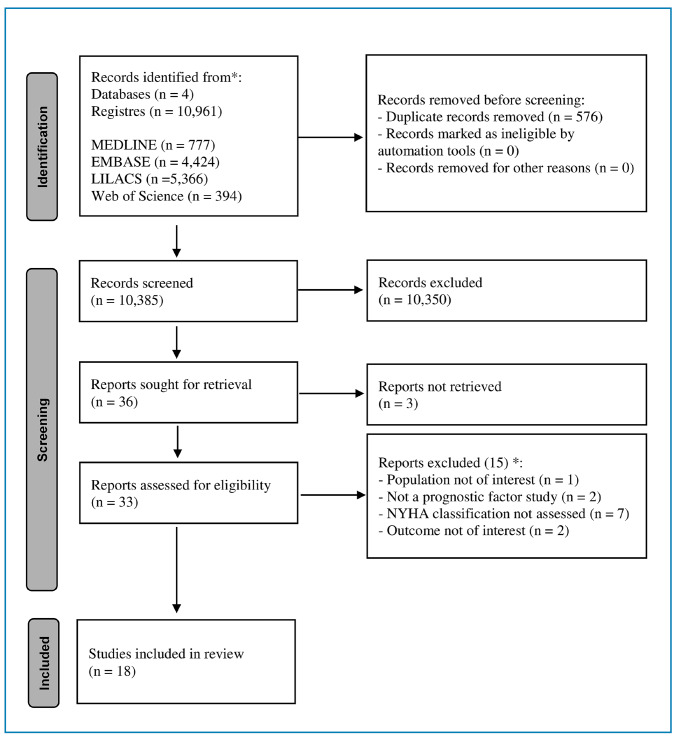
Preferred Reporting Items for Systematic reviews and Meta-Analyses flow diagram of study selection. Articles could be excluded for more than one reason. **NYHA:** New York Heart Association.

Through the electronic search strategy, we identified 10,961 titles and abstracts. Among them, 576 (5.25%) were duplicates. Of the remaining 10,385 references, 36 were selected as potentially eligible studies, and 10,350 were excluded. We did not identify any additional articles for full-text review through our manual search. Finally, 18 studies met our inclusion criteria^17^ ^-^ ^20^ ^,^ ^37^ ^-^ ^50^. Figure 1 outlines the flow of study selection for the systematic review.

**FIGURE 2:**
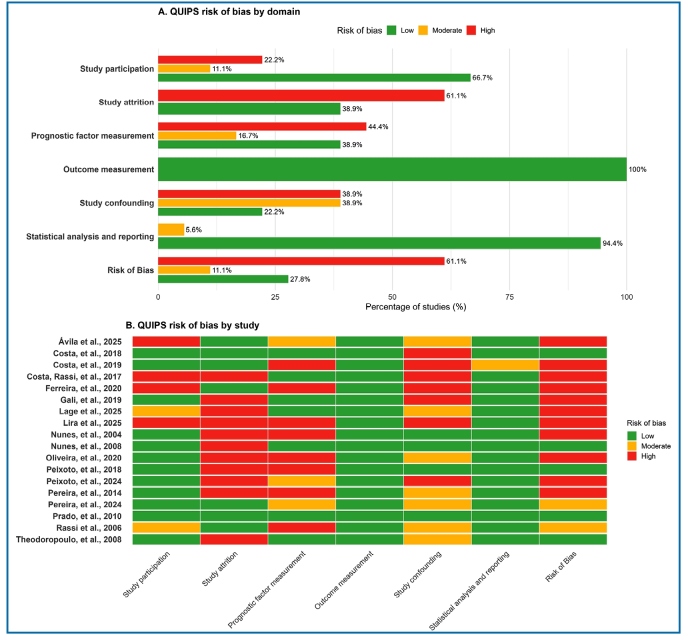
Risk of bias assessment of the included studies using the Quality in Prognostic Studies tool. **(A)** The aggregated risk of bias by QUIPS domain, expressed as the percentage of studies classified as having a low, moderate, or high risk of bias. **(B)** The study-level risk of bias assessment using a traffic-light plot. **QUIP:** Quality in Prognostic Studies.

Of the 18 studies included, 5 (27.77%) had a low risk of bias, 2 (11.11%) had a moderate risk, and 11 (61.11%) had a high risk. Among these 18 studies, 16 involved prospective cohorts, of which 3 were classified as inception cohorts (immediately following exposure to a specific factor), and 2 were classified as retrospective cohorts. The characteristics of the included studies are shown in Table 2. The risk of bias assessment of the included studies, performed using the QUIPS tool, is displayed in Figure 2. The full eligibility assessment and risk of bias evaluation are presented in Supplementary Material Table 1 and Supplementary Material Table 2.

**TABLE 2:** Characteristics of included studies.

Study	Study type	Sample	Prognostic factor	Comparator	Outcome	Follow-up	Prognostic significance and effect estimative	Risk of bias
Ávila et al., 2025	Prospective cohort	Sixty-nine patients with Chagas cardiomyopathy; 41 (59.4%) female; aged 53.70 ± 9.66; LVEF: 47.84 ± 16.6%. NYHA I: n = 43 (62.3%) NYHA II: n = 26 (37.7%)	NYHA functional class (categorical: I-II)	Per one-class increase in NYHA functional class	Composite cardiovascular outcome (cardiac death, heart transplantation, or stroke)	43.81 ± 1.21 months	NYHA: HR = 0.80; CI: 0.27-2.35; p = 0.688*Z	High
Costa et al., 2018	Prospective cohort	Forty-nine patients with Chagas cardiomyopathy; 21 (43%) female; aged 50.0 ± 7.0; LVEF: 36.0% (31.0%-41.0%). NYHA I: n = 28 (57%) NYHA II: n = 16 (33%) NYHA III: n = 5 (10%)	NYHA II and III	NYHA I	Cardiac mortality	39 ± 14 months	NYHA I vs. II and III*: HR = 1.0; CI: 0.2-1.6; p = 0.597	Low
Costa et al., 2019	Prospective cohort	Seventy-five patients with Chagas cardiomyopathy; 64 female; aged 48.4 ± 8.0; LVEF: 41.0% (35.0%-53.5%). NYHA I: n = 45 (60%) NYHA II: n = 24 (32%) NYHA III: n = 6 (8%)	NYHA	Per one-class increase in NYHA functional class	Cardiovascular events, defined as cardiac death, heart transplantation, or ischemic event	41 ± 12 months	NYHA: HR = 0.92; CI: 0.46-1.84; p = 0.823*	High
Costa Rassi et al., 2017	Prospective cohort	Sixty patients with Chagas cardiomyopathy; 24 (45%) female; aged 52.6 ± 9.4; LVEF: 27.5% (25.3%-28.9%). All NYHA II or III	NYHA III	NYHA II	All-cause mortality	7.5 years	NYHA II vs. III: HR = 1.34; CI: 0.76-2.36; p = 0.311	High
Ferreira et al., 2020	Prospective cohort	One-thousand six-hundred thirty-seven patients with Chagas cardiomyopathy; 876 (53.5%) aged over 60; LVEF: not reported. NYHA I: n = 904 (55.7%) NYHA II-IV: n = 718 (44.3%)	NYHA II-IV	NYHA I	Cardiovascular events (characterized as: death from all causes, development of atrial fibrillation, or pacemaker implantation)	2 years	NYHA II-IV: OR = 2.007; CI: 1.402-2.873; p < 0.001	High
Gali et al., 2019	Prospective inception cohort after ICD implantation	Eighty-nine patients with Chagas cardiomyopathy underwent ICD placement; aged 56 ± 11 years; LVEF: 42% ± 12%. NYHA I and II: n = 88 (98%) NYHA III: n = 1 (2%)	NYHA III	NYHA I and II	All-cause mortality or heart transplantation	59 ± 27 months	NYHA III*: HR = 11.69; CI: 1.44-95.00; p = 0.02	Moderate
Lage et al., 2025	Prospective cohort	Five-hundred seventeen patients with Chagas cardiomyopathy; 218 (42%) female; median age 54 (43-62; LVEF: median 39% (29%-50%). NYHA I and II: n = 394 (76%) NYHA III and IV: n = 124 (24%)	NYHA III and IV	NYHA I and II	Occurrence of stroke	4.8 years (IQR, 1.1-7.1	NYHA III-IV: HR = 1.69; CI: 0.80-3.55; p = 0.167	High
Lira et al., 2025	Retrospective cohort	One-hundred seventy-eight patients with Chagas cardiomyopathy; 80 (44.9%) female; median age 60.0 (52.0-67.0); LVEF: median 30.0% (25.0%-35.0%). NYHA I: n = 61 (34.3%) NYHA II: 77 (43.3%) NYHA III: 33 (18.5%) NYHA IV: 7 (3.9%)	NYHA functional class (categorical: I-IV)	Per one-class increase in NYHA functional class	Event-free survival (total mortality and heart transplantation)	15 years	NYHA I-IV: HR = 1,62; CI: 1,19-2,21; p = 0.002	High
Nunes et al., 2004	Prospective cohort	Ninety-three patients with Chagas cardiomyopathy; aged 47.3 ± 12.9. NYHA: 1.8 ± 0.8*	NYHA II and III	NYHA I	Cardiovascular mortality	21.1 ± 10.8 months	NYHA II, III, and IV: RR = 5.02; CI: 1.3-19.8; p = 0.02	High
Nunes et al., 2008	Prospective cohort	One-hundred sixty patients with Chagas cardiomyopathy; aged 48.5 ± 12.3; LVEF: 36.9 ± 12.5%. NYHA I and II: n = 66 (79%) NYHA III and IV: n = 33 (21%)	NYHA III and IV	NYHA I and II	Cardiovascular mortality/sudden death	34 ± 23 months	NYHA III-IV: HR = 2.15; CI: 1.16-4.29; p = 0.030	High
Di Lorenzo Oliveira et al., 2020	Prospective cohort	One-thousand five-hundred eighty-four patients with Chagas cardiomyopathy; aged 59.4 ± 12.7. NYHA I: n = 836 (54%) NYHA II to IV: n = 715 (46%)	NYHA II-IV	NYHA I	All-cause mortality	2 years	NYHA II-IV: HR = 1.628; CI: 1.088-2.437; p = 0.018	High
Peixoto et al., 2018	Prospective cohort	Three-hundred ninety-six patients with Chagas cardiomyopathy and pacemaker; aged 62.5 ± 12.0; LVEF ≤ 43%: 132 (33.3%). NYHA I-II: n = 376 (94.9%) NYHA III-IV: n = 20 (5.1%)	NYHA III-IV	NYHA I and II	All-cause mortality	1.9 years (IQR, 1.6-2.4)	NYHA III-IV: OR = 6.71; CI: 1.95-23.20; p = 0.003	Low
Lima Peixoto et al., 2024	Prospective cohort	Five-hundred fifty-five patients; mean age 63.3 ± 12.0 years. NYHA I-II: n = 528 (95.0%) NYHA III-IV: n = 27 (5.0%)	NYHA III-IV.	NYHA I-II	All-cause mortality	3.7 ± 1.5 years	NYHA III-IV: HR = 2.16; CI: 1.16-4.00; p = 0.014	High
Pereira et al., 2014	Prospective inception cohort after ICD implantation	Sixty-five patients with Chagas cardiomyopathy after ICD implantation; aged 56 ± 11.9; LVEF > 55%: 12 (18.5%), LVEF 45%-55%: 5 (7.7%), LVEF 30%-44%: 15 (23.1%), LVEF < 30%: 33 (50.8%). NYHA I: n = 13 (20%) NYHA II: n = 25 (38.5%) NYHA III: n = 18 (27.5%) NYHA IV: n = 9 (13.8%)	NYHA IV	NYHA I-III	All-cause mortality	40 ± 26.8 months	NYHA IV: HR = 3.5; CI: 0.9-14.4; p = 0.074	High
Pereira et al., 2024	Prospective inception cohort after ICD implantation	One-hundred seventeen patients with Chagas cardiomyopathy after ICD implantation; median age 55 (48-64); LVEF normal: 18 (15.4%), LVEF mild: 6 (13.7%), LVEF moderate: 38 (32.5%), LVEF severe: 45 (38.5%). NYHA I: n = 20 (17.1%) NYHA II: n = 61 (52.1%) NYHA III: n = 29 (24.8%) NYHA IV: n = 7 (6%)	NYHA III	NYHA I-II	All-cause mortality	61 (25-121) months	NYHA III vs. I-II: HR = 1.5; CI: 0.8-3.1; p = 0.21 NYHA IV vs. I-II: HR = 5.4; CI: 1.6-18.5; p = 0.007	Moderate
Prado et al., 2010	Prospective cohort	Ninety-six patients with Chagas cardiomyopathy; median age 57 (IQR, 47.1-61); LVEF > 55%: 12 (18.5%), LVEF 45%-55%: 5 (7.7%), LVEF 30%-44%: 15 (23.1%), LVEF < 30%: 33 (50.8%). NYHA I-II: 34 (35.4%) NYHA III-IV: 62 (64.6%)	NYHA III-IV	NYHA I-II	All-cause mortality	2.3 ± 1.7 years	NYHA III-IV: HR = 2.39; CI: 0.95-6.01	Low
Rassi et al., 2006	Retrospective cohort	Four-hundred twenty-four patients with chronic Chagas cardiomyopathy; aged 47 ± 11. NYHA I-IV: n = 380 (89.6%) NYHA III-IV: n = 44 (10.4%)	NYHA III-IV	NYHA I-II	All-cause mortality	7.9 ± 3.2 years	NYHA III-IV: HR = 4.05; CI: 2.46-6.67; p ≤ 0.001	Moderate
Theodoropoulo et al., 2008	Prospective cohort	One-hundred twenty-seven patients with chronic systolic heart failure secondary to Chagas cardiomyopathy; aged 54 ± 15; LVEF: 33% ± 11%. NYHA IV: 28 (22%)	NYHA IV	NYHA I-III	All-cause mortality	25 ± 19 months	NYHA IV: HR = 1.92; CI: 1.02-3.56; p = 0.034	Low

*Univariate model; mean and standard deviation. Abbreviations: **ICD:** implantable cardioverter-defibrillator; **IQR:** interquartile range; **LVEF:** left ventricular ejection fraction; **NYHA:** New York Heart Association; **RR:** relative risk.

### Mortality

**FIGURE 3:**
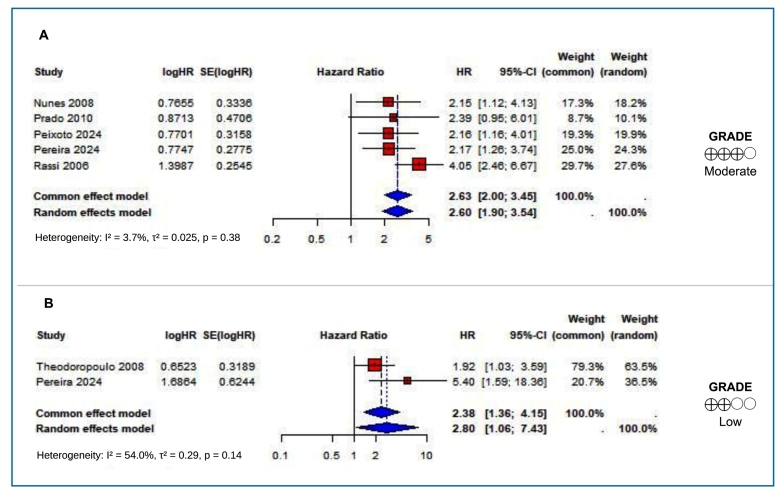
Meta-analyses of the association between the New York Heart Association functional class and mortality outcome. **(A)** Forest plot of the association, expressed as HRs, between exposure to NYHA functional classes II to IV and all-cause mortality in patients with Chagas cardiomyopathy. **(B)** Forest plot of the association, expressed as HRs, between exposure to NYHA functional class IV and all-cause mortality in patients with Chagas cardiomyopathy. **CI:** confidence interval; **HR:** hazard ratio; **NYHA:** New York Heart Association; **SE:** standard error.

Of the seventeen studies in which the prognostic value of the NYHA class on mortality was assessed, six were included in the meta-analyses because they reported comparable mortality outcomes, compatible NYHA exposure and or comparator categories, and adjusted estimates derived from final multivariable models (the descriptions of these studies with the individual estimates are shown in Table 2). The meta-analyses indicated that individuals with NYHA functional classes III and IV had a higher risk of death compared to patients with NYHA classes I and II (hazard ratio [HR] = 2.63; 95% confidence interval [CI]: 2.00-3.45; low statistical heterogeneity [I² = 3.7%; p = 0.38]; moderate quality of evidence; Figure 3) A sensitivity analysis yielded results that were consistent with the primary analysis (HR = 2.70; 95% CI: 1.87-3.91; p < 0.0001; I² = 18.4%; p = 0.29); the study by Lima Peixoto et al.^40^ was excluded from this analysis because it was classified as having a high risk of bias (Supplementary Material Figure 1). Compared to NYHA functional classes I, II, and III, functional class IV was associated with an increased risk of all-cause mortality (HR = 2.80; 95% CI: 1.06-7.43; no statistically significant heterogeneity was observed [I² = 54.0%; p = 0.14]; low quality of evidence).

### Quality of evidence for mortality

The quality of evidence for the prognostic value of the NYHA class on mortality (NYHA classes I and II vs. classes III and IV) was moderate, given serious concerns about publication bias. NYHA class IV compared with NYHA classes I, II, and III was only evaluated in two studies, with the pooled estimate showing high imprecision and serious concerns about publication bias. Therefore, we classified it as having a low quality of evidence. Future research is likely to significantly impact this estimate. The detailed evaluation of the certainty of evidence using the GRADE approach is presented in Supplementary Material Figure 2.

### Cardiovascular events

Cardiovascular outcomes were predominantly evaluated as composite endpoints, with varying combinations of events. In two prospective cohort studies with a high risk of bias^20^ ^,^ ^37^, composite cardiovascular outcomes that included cardiac death, heart transplantation, and stroke or ischemic events were assessed; no association between the NYHA functional class and outcome was found in either of these studies. Similarly, the study by Ferreira et al.^42^, also classified as having a high risk of bias, reported that NYHA functional classes II to IV were associated with a high risk of cardiovascular events; however, this association was observed in a composite outcome in which all-cause mortality, atrial fibrillation, and pacemaker implantation were combined.

Gali et al.^43^ conducted an inception cohort study, which had a moderate risk of bias, and evaluated a composite outcome of all-cause mortality or heart transplantation. They observed an association between NYHA class III and the outcome in univariate analysis, but this association did not persist in the multivariate model. Increasing NYHA functional class was linked to a greater risk of heart transplantation when analyzed within a composite outcome of total mortality and heart transplantation in a retrospective cohort study with a high risk of bias^50^. Conversely, the study by Lage et al.^18^ was the only study in which a cardiovascular outcome alone was assessed; no association between NYHA functional class III or IV and stroke was reported.

Meta-analyses of the prognostic value of the NYHA class on cardiovascular events were not feasible due to heterogeneity across the studies. Particularly, methodological heterogeneity (e.g., differences in study design, population characteristics, and follow-up durations) and variability in NYHA functional class categorization was found.

### Quality of evidence for cardiovascular events

The confidence evidence for cardiovascular events was very low, suggesting that the NYHA class cannot predict heart transplantation or stroke, but it can predict pacemaker implantation. The quality of evidence for cardiovascular outcomes was downgraded to very low because of serious concerns regarding inconsistency, imprecision, risk of bias, and publication bias^36^ ^,^ ^51^.

## DISCUSSION

This was the first systematic review with meta-analysis to assess the prognostic value of the NYHA class in individuals with ChC. Evidence of moderate quality indicated that NYHA classes III and IV predict mortality in this population, whereas evidence warranting low confidence suggests that a NYHA class of IV alone is also associated with mortality. However, evidence warranting very low confidence indicates that the NYHA class cannot predict heart transplantation or stroke, but appears to be capable of predicting implantation of a cardiac pacemaker.

The results of our meta-analyses indicated that, as well as those in an isolated group with class IV (versus those with class I, II, and III), patients with NYHA classes III and IV have a worse prognosis regarding mortality outcomes than those with classes I and II. The NYHA classification is used to assess the symptomatic impact of heart disease on daily activities, ranging from no symptoms (class I) to severe symptoms at rest (class IV)^52^ ^,^ ^53^. In NYHA classes I and II, symptoms are absent or mild when daily activities are performed. Patients with these classes typically present with minimal impairments in functional capacity or cardiac structure and function^53^ ^,^ ^54^. By contrast, patients with classes III and IV exhibit symptoms of heart disease under minimal exertion or even at rest, most often presenting with significant impairments in functional capacity and in cardiac structure and function^53^ ^,^ ^54^. This classification is strongly associated with key markers of disease severity, such as functional capacity^21^, left ventricular ejection fraction^54^, and cardiac fibrosis^55^. This may explain why the class can be to predict mortality outcomes.

Consensus among clinicians and researchers that the NYHA classification is a fundamental tool for assessing the functional impact of heart disease in patients is well established; this is attributed to its ease of application, low cost, and effectiveness in distinguishing degrees of impairment^53^ ^,^ ^56^. However, this review’s findings highlight an additional role for the NYHA classification in predicting mortality: In patients with ChC, well-established prognostic markers such as echocardiographic^57^ and cardiopulmonary exercise testing^58^ variables are often unavailable in endemic regions with low indices for human development^59^. Clinically, this may reduce uncertainty in the use of the NYHA classification as a tool for risk stratification, potentially supporting decision-making regarding closer monitoring and or referral to specialized care when available; particularly, this may support decision-making for access to more complex prognostic tools such as cardiopulmonary exercise testing or echocardiography^58^ ^,^ ^59^.

Three of the four studies in which the prognostic value of the NYHA classification for heart transplantation outcomes were evaluated found no association; however, in two studies, fewer than 10% of the sample were classified as class III or IV. Gali et al.^43^ reported only one patient classified as having NYHA class III; Costa et al.^20^ and Ávila et al.^37^ reported six and none, respectively. Furthermore, none of the studies included patients with NYHA class IV. These findings were contradicted only by the findings of Lira et al.,^50^ who reported a prognostic value of the NYHA class for cardiovascular events; however, this outcome was assessed as a composite that included mortality. Given the methodological and statistical heterogeneity across the studies, this finding should be interpreted with caution, and further studies are warranted.

The small number of patients classified as having NYHA classes III and IV may have limited the number of events observed in the group with greater functional impairment^60^. However, these findings serve as a warning: NYHA class IV-and, in some cases, class III-is an established indication for heart transplantation^61^. This suggests that although patients may meet the criteria for transplantation, the procedure is not being performed. This may be due to patients with ChC, particularly those in endemic regions, having limited access to specialized healthcare services^62^ ^,^ ^63^ as well as the long waiting lists for transplantation^64^ ^-^ ^67^. This underscores the importance of preventing disease progression while patients are still classified as having NYHA classes I and II.

The results show that the prognostic value of the NYHA class for stroke was only evaluated in two studies^18^ ^,^ ^37^. Both studies found no association between NYHA class and stroke incidence. This may be explained by the high propensity for stroke across all stages of ChC, as described in previous studies^68^. This high propensity for stroke to occur, regardless of the severity of the cardiomyopathy, can be explained by the pathophysiology of stroke in ChC, which is closely related to the intense inflammatory process and endothelial dysfunction, common findings throughout the disease cycle^69^. However, this finding must be interpreted with caution, because it was only reported by one study.

Finally, the prognostic power of the NYHA classification in predicting pacemaker implantation was only evaluated in one study, with findings suggesting a potential predictive role^42^. However, the study assessed pacemaker implantation within a composite outcome that included mortality and atrial fibrillation, which may have facilitated statistical significance being achieved because atrial fibrillation can, in some cases, constitute an indication for pacemaker implantation^70^. Therefore, further studies are needed to investigate the ability of the NYHA classification to predict the need for pacemaker implantation.

The results of this systematic review should be interpreted with caution, especially regarding outcomes such as stroke, heart transplantation, and pacemaker implantation, due to the high heterogeneity and limited number of studies included. Furthermore, the unavailability of individual-level data for more detailed analysis was a significant limitation, because it prevented the integration of univariate and multivariate data, extraction at specific time points, and standardization of NYHA functional class categories across studies. Consequently, the pooled comparison of classes III and IV versus classes I and II may have masked potential prognostic gradients across individual NYHA classes. Moreover, conclusions regarding NYHA class IV alone should be interpreted with caution, because this analysis was based on a limited number of studies. Nevertheless, considering their applicability, these findings highlight the NYHA classification as an important and promising variable for risk stratification of patients with ChC. These contributions can enhance decision-making, particularly regarding the frequency of consultations, modification of risk factors through treatment, individualization of care, and overall risk stratification.

## CONCLUSION

The strength of the evidence ranged from very low to moderate. Moderate evidence suggests that NYHA classes III and IV can predict mortality in ChC; however, we have low confidence evidence indicates that NYHA class IV alone may have predictive value for this outcome. Insufficient evidence was available to support a consistent association between the NYHA functional class and heart transplantation or stroke; moreover, this evidence was categorized as very low certainty. However, limited evidence suggested a possible association between the class and pacemaker implantation. Given the high degree of heterogeneity across samples and methods, future research should follow established guidelines for conducting and reporting studies to ensure more robust and comparable results.

## Data Availability

Research data are only available upon request.
