# Supplementary material for: Prognostic value of the New York Heart Association classification for cardiovascular events and mortality in Chagas cardiomyopathy: a systematic review and meta-analysis with GRADE recommendations
Source: Rev Soc Bras Med Trop. 2026 Aug 3;59:e0104-2026. doi: 10.1590/0037-8682-0104-2026 (PMC13432799; doi:10.1590/0037-8682-0104-2026)
Supplement: Supplementary material [file 1678-9849-rsbmt-59-e0104-2026-md1.pdf]

Details in the statistical analysis

We converted the effect estimates and their confidence intervals to the log hazard ratio (log HR) scale to conduct the meta-analyses. This transformation is a recommended step for meta-analysis of prognostic factors [1]. Furthermore, this transformation simplifies the calculation of standard errors and confidence intervals. After performing the meta-analyses, the estimates were converted back to the original scale to facilitate the interpretation of the results [2, 3].

R Scripts for Statistical Analyses

```
library(meta)

estudos <- c( "Nunes 2008", "Prado 2010", "Peixoto 2024", "Pereira 2024", "Rassi 2006")

hr <- c(2.15, 2.39, 2.16, 2.17, 4.05)
ci_lower <- c(1.16, 0.95, 1.16, 1.26, 2.46)
ci_upper <- c(4.29, 6.01, 4.00, 3.74, 6.67)

dados <- data.frame(estudos, hr, ci_lower, ci_upper)

dados$log_hr <- log(dados$hr)
dados$se_log_hr <- (log(dados$ci_upper) - log(dados$ci_lower)) / (2 * 1.96)
metan <- metagen( dados$log_hr, dados$se_log_hr, studlab = dados$estudos, sm = "HR",  method.tau = "REML")

forest(metan,xlab = "Hazard Ratio (HR)", showweights = TRUE, refline = 1)
```

```
library(meta)

estudos <- c("Theodoropoulo 2008", "Pereira 2024")

hr <- c(1.92, 5.40)
ci_lower <- c(1.02, 1.60)
ci_upper <- c(3.56, 18.50)

dados <- data.frame(estudos, hr, ci_lower, ci_upper)

dados$log_hr <- log(dados$hr)
dados$se_log_hr <- (log(dados$ci_upper) - log(dados$ci_lower)) / (2 * 1.96)

metan <- metagen(dados$log_hr, dados$se_log_hr, studlab = dados$estudos,sm = "HR", method.tau = "REML")

forest(metan, xlab = "Hazard Ratio (HR)", showweights = TRUE, refline = 1)
```

Criteria applied in the GRADE assessment

In the adapted GRADE scale for prognostic studies, evidence was rated as high quality (even in observational studies) and downgraded by 1 point for each of the following criteria: risk of bias,

inconsistency, indirectness, imprecision, and publication bias. The confidence of the evidence would increase in the case of moderate (HR of 2 to 4.5) or large (HR more than 4.5) effect sizes and a dose-response gradient [4, 5].

Supplementary Table 1: Study selection according to eligibility criteria.

Study (Author, year)	Population of interest?	Prognostic factor study?	Investigates the predictive value of NYHA?	Outcome of interest?	Included?
Ávila et al., 2022	Y	Y	N	Y	N
Ávila et al., 2025	Y	Y	Y	Y	Y
Bestetti et al., 1994	Y	Y	N	Y	N
Bestetti et al., 2001	Y	Y	Y	N	N
Bestetti et al., 2025	Y	Y	N	Y	N
Cardinalli-Neto et al., 2007	Y	Y	N	Y	N
Cardoso et al., 2016	Y	N	Y	Y	N
Carrasco et al., 1994*	Y	Y	N	Y	N
Costa et al., 2018	Y	Y	Y	Y	Y
Costa et al., 2019	Y	Y	N	Y	Y
Costa & Rassi et al., 2017	Y	Y	Y	Y	Y
Costa, Lima et al., 2017	Y	N	Y	Y	N
Darto et al., 2010	Y	N	N	Y	N
Dib et al., 2009	Y	Y	N	Y	N
Ferreira et al., 2020	Y	Y	Y	Y	Y
Gali et al., 2019	Y	Y	Y	Y	Y
Lage et al., 2025	Y	Y	Y	Y	Y
Lira et al., 2025	Y	Y	Y	Y	Y
Mady et al., 1994	Y	Y	Y	Y	N
Nunes et al., 2004	Y	Y	Y	Y	Y
Nunes et al., 2008	Y	Y	Y	Y	Y
Nunes et al., 2015	Y	N	Y	N	N
Oliveira et al., 2017	Y	N	Y	Y	N
Oliveira et al., 2020	Y	Y	Y	Y	Y
Peixoto et al., 2015	Y	N	Y	Y	N
Peixoto et al., 2015*	Y	N	Y	Y	N
Peixoto et al., 2015*	Y	N	Y	Y	N
Peixoto et al., 2018	Y	Y	Y	Y	Y
Peixoto et al., 2024	Y	Y	Y	Y	Y
Pereira et al., 2014	Y	Y	Y	Y	Y
Pereira et al., 2024	Y	Y	Y	Y	Y
Prado et al., 2010	Y	Y	Y	Y	Y
Sarabanda et al., 2011	Y	Y	N	Y	N
Rassi et al., 2006	Y	Y	Y	Y	Y
Theodoropoulos et al., 2008	Y	Y	Y	Y	Y
Toro et al., 2011	Y	Y	N	Y	N

*: Conference abstract or no full text available; Y: Yes; N: No.

Supplementary Table 2: Detailed QUIPS risk of bias assessments by study.

Study (year)	Study participation	Study attrition	Prognostic factor measurement	Outcome measurement	Study confounding	Statistical analysis and reporting	Risk of Bias
Ávila et al., 2025	High	Low	Moderate	Low	Moderate	Low	High
Costa, et al., 2018	Low	Low	Low	Low	High	Low	Low
Costa, et al., 2019	Low	Low	High	Low	High	Moderate	High
Costa, Rassi, et al., 2017	High	High	Low	Low	High	Low	High
Ferreira, et al., 2020	High	Low	High	Low	High	Low	High
Gali, et al., 2019	Low	High	Low	Low	High	Low	High
Lage et al., 2025	Moderate	High	Low	Low	Moderate	Low	High
Lira et al., 2025	High	High	High	Low	High	Low	High
Nunes, et al., 2004	Low	High	High	Low	Low	Low	High
Nunes, et al., 2008	Low	High	Low	Low	Low	Low	Low
Oliveira, et al., 2020	Low	High	High	Low	Moderate	Low	High
Peixoto, et al., 2018	Low	High	High	Low	Low	Low	Low
Peixoto, et al., 2024	Low	High	Moderate	Low	High	Low	High
Pereira, et al., 2014	Low	High	High	Low	Moderate	Low	High
Pereira, et al., 2024	Low	Low	Moderate	Low	Moderate	Low	Moderate
Prado, et al., 2010	Low	Low	Low	Low	Low	Low	Low
Rassi et al., 2006	Moderate	Low	High	Low	Moderate	Low	Moderate
Theodoropoulo, et al., 2008	Low	High	Low	Low	Moderate	Low	Low

Supplementary Figure 1: Sensitivity analysis of the association between NYHA functional class and all-cause mortality after exclusion of the study classified as high risk of bias (Peixoto *et al*., 2024).

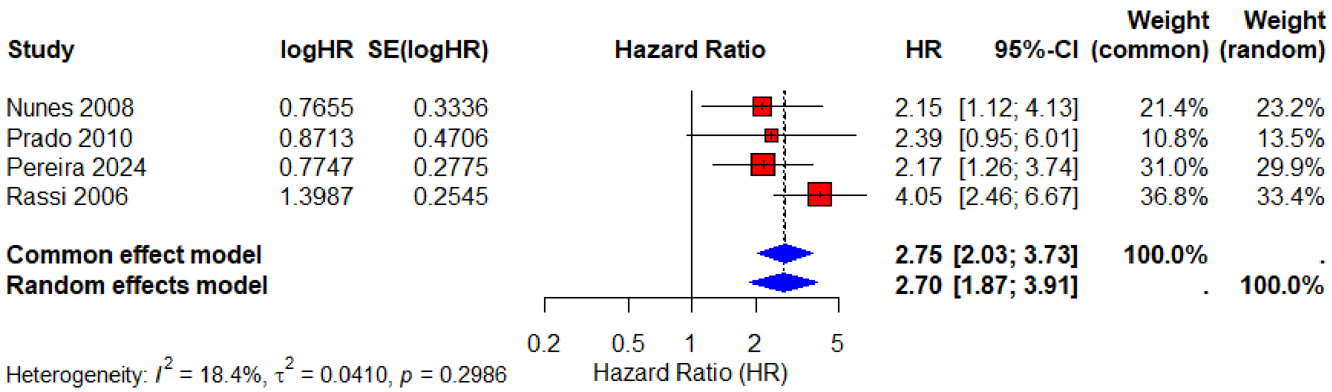

Supplementary Figure 2: GRADE assessment of evidence confidence.

Number of studies	Study design / Limitations	Risk of bias	Inconsistency	Indirectness	Imprecision	Publication bias	Other considerations	HR (95 % CI)	Overall Quality
**Mortality (NYHA III to IV vs I and II)**									
5	Cohorts	Not serious	Serious	Not serious	Not serious	Serious	None	HR* of 2.63 (95% CI: 2.00–3.45)	⊕⊕⊕◯ - Moderate
**Mortality (NYHA IV vs. I, II, and III)**									
2	Cohorts	Not serious	Not serious	Not serious	Serious	Serious	None	HR of 2.38 (95% CI: 1.36–4.15)	⊕⊕◯◯ - Low

*Evidence was downgraded for inconsistency due to heterogeneity in study populations and analytical approaches. However, no downgrading was applied for imprecision given the narrow confidence interval and the robust magnitude of the observed effect (HR >2).

Search strategy

Search	Query	Records retrieved
**MEDLINE**	Journals@Ovid Full Text <January 16, 2026><br>Your Journals@Ovid<br>Biological Abstracts <1999 to 2014><br>Ovid MEDLINE(R) and Epub Ahead of Print, In-Process, In-Data-Review & Other Non-Indexed Citations, Daily and Versions <1946 to January 16, 2026><br><br>1 Validat$.mp. [mp=ti, ab, tx, ct, mc, st, or, tn, ps, ds, cb, rn, sq, mq, ge, tm, mi, sh, bo, bt, hw, ot, nm, fx, kf, ox, px, rx, an, ui, sy, ux, mx]<br>2 Predict$.ti.<br>3 Rule$.mp. [mp=ti, ab, tx, ct, mc, st, or, tn, ps, ds, cb, rn, sq, mq, ge, tm, mi, sh, bo, bt, hw, ot, nm, fx, kf, ox, px, rx, an, ui, sy, ux, mx]<br>4 1 or 2 or 3<br>5 Predict$.mp. [mp=ti, ab, tx, ct, mc, st, or, tn, ps, ds, cb, rn, sq, mq, ge, tm, mi, sh, bo, bt, hw, ot, nm, fx, kf, ox, px, rx, an, ui, sy, ux, mx]<br>6 Outcome$.mp. [mp=ti, ab, tx, ct, mc, st, or, tn, ps, ds, cb, rn, sq, mq, ge, tm, mi, sh, bo, bt, hw, ot, nm, fx, kf, ox, px, rx, an, ui, sy, ux, mx]<br>7 Risk$.mp. [mp=ti, ab, tx, ct, mc, st, or, tn, ps, ds, cb, rn, sq, mq, ge, tm, mi, sh, bo, bt, hw, ot, nm, fx, kf, ox, px, rx, an, ui, sy, ux, mx]<br>8 Model$.mp. [mp=ti, ab, tx, ct, mc, st, or, tn, ps, ds, cb, rn, sq, mq, ge, tm, mi, sh, bo, bt, hw, ot, nm, fx, kf, ox, px, rx, an, ui, sy, ux, mx]<br>9 6 or 7 or 8<br>10 5 and 9<br>11 History.mp. [mp=ti, ab, tx, ct, mc, st, or, tn, ps, ds, cb, rn, sq, mq, ge, tm, mi, sh, bo, bt, hw, ot, nm, fx, kf, ox, px, rx, an, ui, sy, ux, mx]	777

12 Variable$.mp. [mp=ti, ab, tx, ct, mc, st, or, tn, ps, ds, cb, rn, sq, mq, ge, tm, mi, sh, bo, bt, hw, ot, nm, fx, kf, ox, px, rx, an, ui, sy, ux, mx]
13 Criteria.mp. [mp=ti, ab, tx, ct, mc, st, or, tn, ps, ds, cb, rn, sq, mq, ge, tm, mi, sh, bo, bt, hw, ot, nm, fx, kf, ox, px, rx, an, ui, sy, ux, mx]
14 Scor$.mp. [mp=ti, ab, tx, ct, mc, st, or, tn, ps, ds, cb, rn, sq, mq, ge, tm, mi, sh, bo, bt, hw, ot, nm, fx, kf, ox, px, rx, an, ui, sy, ux, mx]
15 Characteristic$.mp. [mp=ti, ab, tx, ct, mc, st, or, tn, ps, ds, cb, rn, sq, mq, ge, tm, mi, sh, bo, bt, hw, ot, nm, fx, kf, ox, px, rx, an, ui, sy, ux, mx]
16 Finding$.mp. [mp=ti, ab, tx, ct, mc, st, or, tn, ps, ds, cb, rn, sq, mq, ge, tm, mi, sh, bo, bt, hw, ot, nm, fx, kf, ox, px, rx, an, ui, sy, ux, mx]
17 Factor$.mp. [mp=ti, ab, tx, ct, mc, st, or, tn, ps, ds, cb, rn, sq, mq, ge, tm, mi, sh, bo, bt, hw, ot, nm, fx, kf, ox, px, rx, an, ui, sy, ux, mx]
18 11 or 12 or 13 or 14 or 15 or 16 or 17
19 Predict$.mp. [mp=ti, ab, tx, ct, mc, st, or, tn, ps, ds, cb, rn, sq, mq, ge, tm, mi, sh, bo, bt, hw, ot, nm, fx, kf, ox, px, rx, an, ui, sy, ux, mx]
20 Model$.mp. [mp=ti, ab, tx, ct, mc, st, or, tn, ps, ds, cb, rn, sq, mq, ge, tm, mi, sh, bo, bt, hw, ot, nm, fx, kf, ox, px, rx, an, ui, sy, ux, mx]
21 Decision$.mp. [mp=ti, ab, tx, ct, mc, st, or, tn, ps, ds, cb, rn, sq, mq, ge, tm, mi, sh, bo, bt, hw, ot, nm, fx, kf, ox, px, rx, an, ui, sy, ux, mx]
22 Identif$.mp. [mp=ti, ab, tx, ct, mc, st, or, tn, ps, ds, cb, rn, sq, mq, ge, tm, mi, sh, bo, bt, hw, ot, nm, fx, kf, ox, px, rx, an, ui, sy, ux, mx]
23 Prognos$.mp. [mp=ti, ab, tx, ct, mc, st, or, tn, ps, ds, cb, rn, sq, mq, ge, tm, mi, sh, bo, bt, hw, ot, nm, fx, kf, ox, px, rx, an, ui, sy, ux, mx]
24 19 or 20 or 21 or 22 or 23
25 Decision$.mp. [mp=ti, ab, tx, ct, mc, st, or, tn, ps, ds, cb, rn, sq, mq, ge, tm, mi, sh, bo, bt, hw, ot, nm, fx, kf, ox, px, rx, an, ui, sy, ux, mx]
26 Model$.mp. [mp=ti, ab, tx, ct, mc, st, or, tn, ps, ds, cb, rn, sq, mq, ge, tm, mi, sh, bo, bt, hw, ot, nm, fx, kf, ox, px, rx, an, ui, sy, ux, mx]
27 Clinical$.mp. [mp=ti, ab, tx, ct, mc, st, or, tn, ps, ds, cb, rn, sq, mq, ge, tm, mi, sh, bo, bt, hw, ot, nm, fx, kf, ox, px, rx, an, ui, sy, ux, mx]
28 Logistic Models/
29 26 or 27 or 28
30 25 and 29
31 Prognostic.mp. [mp=ti, ab, tx, ct, mc, st, or, tn, ps, ds, cb, rn, sq, mq, ge, tm, mi, sh, bo, bt, hw, ot, nm, fx, kf, ox, px, rx, an, ui, sy, ux, mx]
32 History.mp. [mp=ti, ab, tx, ct, mc, st, or, tn, ps, ds, cb, rn, sq, mq, ge, tm, mi, sh, bo, bt, hw, ot, nm, fx, kf, ox, px, rx, an, ui, sy, ux, mx]
33 Variable$.mp. [mp=ti, ab, tx, ct, mc, st, or, tn, ps, ds, cb, rn, sq, mq, ge, tm, mi, sh, bo, bt, hw, ot, nm, fx, kf, ox, px, rx, an, ui, sy, ux, mx]
34 Criteria.mp. [mp=ti, ab, tx, ct, mc, st, or, tn, ps, ds, cb, rn, sq, mq, ge, tm, mi, sh, bo, bt, hw, ot, nm, fx, kf, ox, px, rx, an, ui, sy, ux, mx]
35 Scor$.mp. [mp=ti, ab, tx, ct, mc, st, or, tn, ps, ds, cb, rn, sq, mq, ge, tm, mi, sh, bo, bt, hw, ot, nm, fx, kf, ox, px, rx, an, ui, sy, ux, mx]
36 Characteristic$.mp. [mp=ti, ab, tx, ct, mc, st, or, tn, ps, ds, cb, rn, sq, mq, ge, tm, mi, sh, bo, bt, hw, ot, nm, fx, kf, ox, px, rx, an, ui, sy, ux, mx]
37 Finding$.mp. [mp=ti, ab, tx, ct, mc, st, or, tn, ps, ds, cb, rn, sq, mq, ge, tm, mi, sh, bo, bt, hw, ot, nm, fx, kf, ox, px, rx, an, ui, sy, ux, mx]
38 Factor$.mp. [mp=ti, ab, tx, ct, mc, st, or, tn, ps, ds, cb, rn, sq, mq, ge, tm, mi, sh, bo, bt, hw, ot, nm, fx, kf, ox, px, rx, an, ui, sy, ux, mx]
39 Model$.mp. [mp=ti, ab, tx, ct, mc, st, or, tn, ps, ds, cb, rn, sq, mq, ge, tm, mi, sh, bo, bt, hw, ot, nm, fx, kf, ox, px, rx, an, ui, sy, ux, mx]
40 32 or 33 or 34 or 35 or 36 or 37 or 38 or 39
41 31 and 40
42 4 or 10 or 18
43 24 or 30 or 40
44 41 and 42 and 43
45 Chagas' Disease.mp. [mp=ti, ab, tx, ct, mc, st, or, tn, ps, ds, cb, rn, sq, mq, ge, tm, mi, sh, bo, bt, hw, ot, nm, fx, kf, ox, px, rx, an, ui, sy, ux, mx]
46 Chagas cardiomyopat*.mp. [mp=ti, ab, tx, ct, mc, st, or, tn, ps, ds, cb, rn, sq, mq, ge, tm, mi, sh, bo, bt, hw, ot, nm, fx, kf, ox, px, rx, an, ui, sy, ux, mx]

	47 Chagas' Cardiomyopathy.mp. [mp=ti, ab, tx, ct, mc, st, or, tn, ps, ds, cb, rn, sq, mq, ge, tm, mi, sh, bo, bt, hw, ot, nm, fx, kf, ox, px, rx, an, ui, sy, ux, mx]<br>48 Myocarditis, Chagas.mp. [mp=ti, ab, tx, ct, mc, st, or, tn, ps, ds, cb, rn, sq, mq, ge, tm, mi, sh, bo, bt, hw, ot, nm, fx, kf, ox, px, rx, an, ui, sy, ux, mx]<br>49 Chagas heart disease.mp. [mp=ti, ab, tx, ct, mc, st, or, tn, ps, ds, cb, rn, sq, mq, ge, tm, mi, sh, bo, bt, hw, ot, nm, fx, kf, ox, px, rx, an, ui, sy, ux, mx]<br><br>50 American trypanosomia*.mp. [mp=ti, ab, tx, ct, mc, st, or, tn, ps, ds, cb, rn, sq, mq, ge, tm, mi, sh, bo, bt, hw, ot, nm, fx, kf, ox, px, rx, an, ui, sy, ux, mx]<br>51 Chagas myocardiopathy.mp. [mp=ti, ab, tx, ct, mc, st, or, tn, ps, ds, cb, rn, sq, mq, ge, tm, mi, sh, bo, bt, hw, ot, nm, fx, kf, ox, px, rx, an, ui, sy, ux, mx]<br>52 Chagas myocarditis.mp. [mp=ti, ab, tx, ct, mc, st, or, tn, ps, ds, cb, rn, sq, mq, ge, tm, mi, sh, bo, bt, hw, ot, nm, fx, kf, ox, px, rx, an, ui, sy, ux, mx]<br><br>53 Chronic chagas disease cardiomyopathy.mp. [mp=ti, ab, tx, ct, mc, st, or, tn, ps, ds, cb, rn, sq, mq, ge, tm, mi, sh, bo, bt, hw, ot, nm, fx, kf, ox, px, rx, an, ui, sy, ux, mx]<br>54 45 or 46 or 47 or 48 or 49 or 50 or 51 or 52 or 53<br>55 Mortalit*.mp. [mp=ti, ab, tx, ct, mc, st, or, tn, ps, ds, cb, rn, sq, mq, ge, tm, mi, sh, bo, bt, hw, ot, nm, fx, kf, ox, px, rx, an, ui, sy, ux, mx]<br>56 Case fatality rate.mp. [mp=ti, ab, tx, ct, mc, st, or, tn, ps, ds, cb, rn, sq, mq, ge, tm, mi, sh, bo, bt, hw, ot, nm, fx, kf, ox, px, rx, an, ui, sy, ux, mx]<br>57 Death rate.mp. [mp=ti, ab, tx, ct, mc, st, or, tn, ps, ds, cb, rn, sq, mq, ge, tm, mi, sh, bo, bt, hw, ot, nm, fx, kf, ox, px, rx, an, ui, sy, ux, mx]<br>58 Mortality rate*.mp. [mp=ti, ab, tx, ct, mc, st, or, tn, ps, ds, cb, rn, sq, mq, ge, tm, mi, sh, bo, bt, hw, ot, nm, fx, kf, ox, px, rx, an, ui, sy, ux, mx]<br>59 Stroke*.mp. [mp=ti, ab, tx, ct, mc, st, or, tn, ps, ds, cb, rn, sq, mq, ge, tm, mi, sh, bo, bt, hw, ot, nm, fx, kf, ox, px, rx, an, ui, sy, ux, mx]<br>60 Cerebrovascular Accident*.mp. [mp=ti, ab, tx, ct, mc, st, or, tn, ps, ds, cb, rn, sq, mq, ge, tm, mi, sh, bo, bt, hw, ot, nm, fx, kf, ox, px, rx, an, ui, sy, ux, mx]<br>61 Cerebrovascular apoplexy.mp. [mp=ti, ab, tx, ct, mc, st, or, tn, ps, ds, cb, rn, sq, mq, ge, tm, mi, sh, bo, bt, hw, ot, nm, fx, kf, ox, px, rx, an, ui, sy, ux, mx]<br>62 Brain Vascular Accident*.mp. [mp=ti, ab, tx, ct, mc, st, or, tn, ps, ds, cb, rn, sq, mq, ge, tm, mi, sh, bo, bt, hw, ot, nm, fx, kf, ox, px, rx, an, ui, sy, ux, mx]<br>63 Grafting, heart.mp. [mp=ti, ab, tx, ct, mc, st, or, tn, ps, ds, cb, rn, sq, mq, ge, tm, mi, sh, bo, bt, hw, ot, nm, fx, kf, ox, px, rx, an, ui, sy, ux, mx]<br>64 Heart graft*.mp. [mp=ti, ab, tx, ct, mc, st, or, tn, ps, ds, cb, rn, sq, mq, ge, tm, mi, sh, bo, bt, hw, ot, nm, fx, kf, ox, px, rx, an, ui, sy, ux, mx] 4488<br>65 Heart transplantati*.mp. [mp=ti, ab, tx, ct, mc, st, or, tn, ps, ds, cb, rn, sq, mq, ge, tm, mi, sh, bo, bt, hw, ot, nm, fx, kf, ox, px, rx, an, ui, sy, ux, mx]<br>66 Transplantations, heart.mp. [mp=ti, ab, tx, ct, mc, st, or, tn, ps, ds, cb, rn, sq, mq, ge, tm, mi, sh, bo, bt, hw, ot, nm, fx, kf, ox, px, rx, an, ui, sy, ux, mx]<br>67 Cardiac transplantat*.mp. [mp=ti, ab, tx, ct, mc, st, or, tn, ps, ds, cb, rn, sq, mq, ge, tm, mi, sh, bo, bt, hw, ot, nm, fx, kf, ox, px, rx, an, ui, sy, ux, mx]<br><br>68 Artificial pacemaker.mp. [mp=ti, ab, tx, ct, mc, st, or, tn, ps, ds, cb, rn, sq, mq, ge, tm, mi, sh, bo, bt, hw, ot, nm, fx, kf, ox, px, rx, an, ui, sy, ux, mx]<br>69 Pacemaker.mp. [mp=ti, ab, tx, ct, mc, st, or, tn, ps, ds, cb, rn, sq, mq, ge, tm, mi, sh, bo, bt, hw, ot, nm, fx, kf, ox, px, rx, an, ui, sy, ux, mx]<br>70 Pacemaker implant*.mp. [mp=ti, ab, tx, ct, mc, st, or, tn, ps, ds, cb, rn, sq, mq, ge, tm, mi, sh, bo, bt, hw, ot, nm, fx, kf, ox, px, rx, an, ui, sy, ux, mx]<br>71 55 or 56 or 57 or 58 or 59 or 60 or 61 or 62 or 63 or 64 or 65 or 66 or 67 or 68 or 69 or 70<br>72 44 and 54 and 71	
**EMBASE**	# 1 'chagas disease'/exp OR 'american trypanosomiasis' OR 'chagas disease' OR 'chagas infection' OR 'chagas mazza disease' OR 'chagas`s disease' OR	4,424

	'trypanosoma cruzi infection' OR 'disease, chagas' OR 'infection by trypanosoma cruzi' OR 'infection of trypanosoma cruzi' OR 'south american trypanosomiasis' OR 'trypanosomiasis, american' OR 'trypanosomiasis, south american' OR 'chagas cardiomyopathy'/exp OR 'chagas cardiomyopathy' OR 'chagas disease cardiomyopathy' OR 'chagas heart disease' OR 'chagas myocardiopathy' OR 'chagas myocarditis' OR 'cardiomyopathy in chagas disease' OR 'chronic chagas disease cardiomyopathy'<br># 2 'case fatality rate' OR 'mortality'/exp OR 'mortality' OR 'mortality model' OR 'mortality rate'/exp OR 'death rate' OR 'death rate model' OR 'fatal outcome rate' OR 'fatality rate' OR 'lethal outcome rate' OR 'mortality rate' OR 'rate, mortality' OR 'death'/exp OR 'death' OR 'mors' OR 'cerebrovascular accident'/exp OR 'cva' OR 'accident, cerebrovascular' OR 'acute cerebrovascular lesion' OR 'acute focal cerebral vasculopathy' OR 'acute stroke' OR 'apoplectic stroke' OR 'apoplexia' OR 'apoplexy' OR 'blood flow disturbance, brain' OR 'brain accident' OR 'brain attack' OR 'brain blood flow disturbance' OR 'brain insult' OR 'brain insultus' OR 'brain vascular accident' OR 'cerebral apoplexia' OR 'cerebral insult' OR 'cerebral stroke' OR 'cerebral vascular accident' OR 'cerebral vascular insufficiency' OR 'cerebro vascular accident' OR 'cerebrovascular accident' OR 'cerebrovascular arrest' OR 'cerebrovascular failure' OR 'cerebrovascular injury' OR 'cerebrovascular insufficiency' OR 'cerebrovascular insult' OR 'cerebrum vascular accident' OR 'cryptogenic stroke' OR 'insultus cerebralis' OR 'ischaemic seizure' OR 'ischemic seizure' OR 'stroke' OR 'thrombotic stroke' OR 'heart transplantation'/exp OR 'cardiac transplantation' OR 'heart allograft' OR 'heart allotransplantation' OR 'heart heterograft' OR 'heart heterotransplantation' OR 'heart homograft' OR 'heart homotransplantation' OR 'heart orthotopic transplantation' OR 'heart tissue transplantation' OR 'heart transplantation' OR 'heart ventricle transplantation' OR 'human heart transplantation' OR 'transplantation, heart' OR 'pacemaker electrode'/exp OR 'attain ability' OR 'attain ability plus' OR 'attain ability straight' OR 'attain performa' OR 'attain performa s' OR 'attain performa straight' OR 'attain stability quad mri surescan' OR 'attain starfix' OR 'beflex (pacemaker electrode)' OR 'bipolar capsureepi is 1' OR 'corox' OR 'easytrak 2' OR 'endotak reliance g' OR 'flextend' OR 'isoflex 1642t' OR 'linox smart' OR 'linoxsmart' OR 'medtronic 5071' OR 'medtronic 5076' OR 'myodex' OR 'myopore' OR 'safio s' OR 'selectsecure' OR 'sentus promri otw' OR 'solia s' OR 'sprint fidelis' OR 'sprint quattro' OR 'starfix' OR 'unipolar transvene svc model 6937' OR 'vega (pacemaker electrode)' OR 'xfine (pacemaker electrode)' OR 'cardiac pacemaker electrode' OR 'cardiac pacemaker lead' OR 'coronary venous pacing lead' OR 'electrode, pacemaker' OR 'electrode, pacing' OR 'lead, pacemaker' OR 'pacemaker electrode' OR 'pacemaker lead' OR 'pacing electrode' OR 'venous pacing lead'<br># 3 #1 AND #2	
**LILACS**	(Doença de Chagas) OR (Chagas Disease) OR (Enfermedad de Chagas) OR (Maladie de Chagas) OR (Infecção por Trypanosoma cruzi) OR (Mal de Chagas) OR (Tripanossomose Sul-Americana) OR (Tripanossomíase Americana) OR (Tripanossomíase Sul-Americana) AND (Registros de Mortalidade) OR (Sistema de Informações sobre Mortalidade) OR (Mortality Registries) OR (Registros de Mortalidad) OR (Registres de Mortalité) OR (Índice de Casos Fatais) OR (Índice de Fatalidade) OR (Índice de Letalidade) OR (Índice de Mortalidade) OR (Letalidade) OR (Mortality Declines) OR (Mortality Rate) OR (Mortality Rates) OR (Transplante de Coração) OR (Heart Transplantation) OR (Trasplante de Corazón) OR (Transplantation cardiaque) OR (Enxerto Cardíaco) OR (Enxerto de Coração) OR (Transplantação Cardíaco) OR (Transplantação de Coração) OR (Transplante Cardíaco) OR (Marca-Passo Artificial) OR (Artificial Cardiac Pacemaker) OR (Artificial Cardiac Pacemakers) OR (Artificial Pacemakers) OR (Marcapaso Artificial) OR (Pacemaker) OR (AVC Trombótico) OR (AVC Trombótico Agudo) OR (Acute Thrombotic Stroke) OR (Acute Thrombotic Strokes) OR (Stroke, Acute Thrombotic) OR (Stroke, Thrombotic) OR (Thrombotic Strokes) OR (Acute Ischemic Stroke) OR (Acute Ischemic Strokes) OR (Ischaemic Stroke)	5,366
**Web of Science**	Tópico - ("American Trypanosomiasis" OR "Trypanosomiasis, American" OR "Trypanosomiasis, South American" OR "South American Trypanosomiasis"	394

	OR "Trypanosoma cruzi Infection" OR "Infection, Trypanosoma cruzi" OR “Infections, Trypanosoma cruzi” OR “Trypanosoma cruzi Infections” OR “Chagas' Disease” OR “Cardiomyopathy, Chagas” OR “Trypanosomiasis, Cardiovascular” OR “Cardiovascular Trypanosomiasis” OR “Chagas' Cardiomyopathy” OR “Cardiomyopathy, Chagas'” OR “Myocarditis, Chagas”)) AND ALL=(“Mortalities” OR “Case Fatality Rate” OR “Case Fatality Rates” OR “Rate, Case Fatality” OR “Rates, Case Fatality” OR “CFR Case Fatality Rate” OR “Crude Death Rate” OR “Crude Death Rates” OR “Death Rate, Crude” OR “Rate, Crude Death” OR “Crude Mortality Rate” OR “Crude Mortality Rates” OR “Mortality Rate, Crude” OR “Rate, Crude Mortality” OR “Death Rate” OR “Death Rates” OR “Rate, Death” OR “Mortality Rate” OR “Mortality Rates” OR “Rate, Mortality” OR “Mortality, Excess” OR “Excess Mortality” OR “Excess Mortalities” OR “Decline, Mortality” OR “Mortality Declines” OR “Mortality Decline” OR “Mortality Determinants” OR “Age-Specific Death Rate” OR “Age-Specific Death Rates” OR “Death Rate, Age-Specific” OR “Rate, Age-Specific Death” OR “Strokes” OR “Cerebrovascular Accident” OR “Cerebrovascular Accidents” OR ”Vascular Accident, Brain” OR “Brain Vascular Accident” OR “Heart Transplantations” OR “Transplantations, Heart” OR “Cardiac Transplantation” OR “Cardiac Transplantations” OR “Transplantations, Cardiac” OR “Transplantation, Cardiac” OR “Artificial Pacemaker” OR “Artificial Pacemakers” OR “Pacemakers, Artificial”	
**Total**		10,961

Search strategy conducted in the databases with limits and filters, when used. Literature updates were monitored by email alerts. Search conducted on January 18, 2023, and updated on January 20, 2026. Strategy reported according to PRISMA-S: an extension to the PRISMA Statement for Reporting Literature Searches in Systematic Reviews.

*The terms for the search strategy related to the prognostic study were those recommended by the Cochrane Prognosis Methods Group (sensitivity of 0.98 and specificity of 0.96) [6]

REFERENCES

[1] Borenstein M, Hedges LV, Higgins JP, Rothstein HR. Introduction to meta-analysis: John Wiley & Sons; 2021.

[2] Bland JM, Altman DG. The odds ratio. Bmj. 2000;320:1468.

[3] Schwarzer G, Carpenter JR, Rücker G. Meta-analysis with R: Springer; 2015.

[4] Huguet A, Hayden JA, Stinson J, McGrath PJ, Chambers CT, Tougas ME, et al. Judging the quality of evidence in reviews of prognostic factor research: adapting the GRADE framework. Systematic reviews. 2013;2:1-12.

[5] Iorio A, Spencer FA, Falavigna M, Alba C, Lang E, Burnand B, et al. Use of GRADE for assessment of evidence about prognosis: rating confidence in estimates of event rates in broad categories of patients. bmj. 2015;350.

[6] Ingui BJ, Rogers MA. Searching for clinical prediction rules in MEDLINE. Journal of the American Medical Informatics Association. 2001;8:391-7.
