# Supplementary material for: Prognostic value of the New York Heart Association classification for cardiovascular events and mortality in Chagas cardiomyopathy: a systematic review and meta-analysis with GRADE recommendations
Source: Rev Soc Bras Med Trop. 2026 Aug 3;59:e0104-2026. doi: 10.1590/0037-8682-0104-2026 (PMC13432799; doi:10.1590/0037-8682-0104-2026)
Supplement: Supplementary material Table 1 [file 1678-9849-rsbmt-59-e0104-2026-md2.pdf]

Supplementary Table 1: Study selection according to eligibility criteria.

Study (Author, year)	Population of interest?	Prognostic factor study?	Investigates the predictive value of NYHA?	Outcome of interest?	Included?
Ávila et al., 2022	Y	Y	N	Y	N
Ávila et al., 2025	Y	Y	Y	Y	Y
Bestetti et al., 1994	Y	Y	N	Y	N
Bestetti et al., 2001	Y	Y	Y	N	N
Bestetti et al., 2025	Y	Y	N	Y	N
Cardinalli-Neto et al., 2007	Y	Y	N	Y	N
Cardoso et al., 2016	Y	N	Y	Y	N
Carrasco et al., 1994*	Y	Y	N	Y	N
Costa et al., 2018	Y	Y	Y	Y	Y
Costa et al., 2019	Y	Y	N	Y	Y
Costa & Rassi et al., 2017	Y	Y	Y	Y	Y
Costa, Lima et al., 2017	Y	N	Y	Y	N
Darto et al., 2010	Y	N	N	Y	N
Dib et al., 2009	Y	Y	N	Y	N
Ferreira et al., 2020	Y	Y	Y	Y	Y
Gali et al., 2019	Y	Y	Y	Y	Y
Lage et al., 2025	Y	Y	Y	Y	Y
Lira et al., 2025	Y	Y	Y	Y	Y
Mady et al., 1994	Y	Y	Y	Y	N
Nunes et al., 2004	Y	Y	Y	Y	Y
Nunes et al., 2008	Y	Y	Y	Y	Y
Nunes et al., 2015	Y	N	Y	N	N
Oliveira et al., 2017	Y	N	Y	Y	N
Oliveira et al., 2020	Y	Y	Y	Y	Y
Peixoto et al., 2015	Y	N	Y	Y	N
Peixoto et al., 2015*	Y	N	Y	Y	N
Peixoto et al., 2015*	Y	N	Y	Y	N
Peixoto et al., 2018	Y	Y	Y	Y	Y
Peixoto et al., 2024	Y	Y	Y	Y	Y
Pereira et al., 2014	Y	Y	Y	Y	Y
Pereira et al., 2024	Y	Y	Y	Y	Y
Prado et al., 2010	Y	Y	Y	Y	Y
Sarabanda et al., 2011	Y	Y	N	Y	N
Rassi et al., 2006	Y	Y	Y	Y	Y
Theodoropoulos et al., 2008	Y	Y	Y	Y	Y
Toro et al., 2011	Y	Y	N	Y	N

*: Conference abstract or no full text available; Y: Yes; N: No.
