# Supplementary material for: Prognostic value of the New York Heart Association classification for cardiovascular events and mortality in Chagas cardiomyopathy: a systematic review and meta-analysis with GRADE recommendations
Source: Rev Soc Bras Med Trop. 2026 Aug 3;59:e0104-2026. doi: 10.1590/0037-8682-0104-2026 (PMC13432799; doi:10.1590/0037-8682-0104-2026)
Supplement: Supplementary material Table 2 [file 1678-9849-rsbmt-59-e0104-2026-md3.pdf]

Revista da Sociedade Brasileira de Medicina Tropical
Journal of the Brazilian Society of Tropical Medicine

Supplementary Table 2: Detailed QUIPS risk of bias assessments by study.

Study (year)	Study participation	Study attrition	Prognostic factor measurement	Outcome measurement	Study confounding	Statistical analysis and reporting	Risk of Bias
Ávila et al., 2025	High	Low	Moderate	Low	Moderate	Low	High
Costa, et al., 2018	Low	Low	Low	Low	High	Low	Low
Costa, et al., 2019	Low	Low	High	Low	High	Moderate	High
Costa, Rassi, et al., 2017	High	High	Low	Low	High	Low	High
Ferreira, et al., 2020	High	Low	High	Low	High	Low	High
Gali, et al., 2019	Low	High	Low	Low	High	Low	High
Lage et al., 2025	Moderate	High	Low	Low	Moderate	Low	High
Lira et al., 2025	High	High	High	Low	High	Low	High
Nunes, et al., 2004	Low	High	High	Low	Low	Low	High
Nunes, et al., 2008	Low	High	Low	Low	Low	Low	Low
Oliveira, et al., 2020	Low	High	High	Low	Moderate	Low	High
Peixoto, et al., 2018	Low	High	High	Low	Low	Low	Low
Peixoto, et al., 2024	Low	High	Moderate	Low	High	Low	High
Pereira, et al., 2014	Low	High	High	Low	Moderate	Low	High
Pereira, et al., 2024	Low	Low	Moderate	Low	Moderate	Low	Moderate
Prado, et al., 2010	Low	Low	Low	Low	Low	Low	Low
Rassi et al., 2006	Moderate	Low	High	Low	Moderate	Low	Moderate
Theodoropoulo, et al., 2008	Low	High	Low	Low	Moderate	Low	Low
