# Supplementary material for: Prognostic value of the New York Heart Association classification for cardiovascular events and mortality in Chagas cardiomyopathy: a systematic review and meta-analysis with GRADE recommendations
Source: Rev Soc Bras Med Trop. 2026 Aug 3;59:e0104-2026. doi: 10.1590/0037-8682-0104-2026 (PMC13432799; doi:10.1590/0037-8682-0104-2026)

Supplementary Figure 1: Sensitivity analysis of the association between NYHA functional class and all-cause mortality after exclusion of the study classified as high risk of bias (Peixoto *et al.*, 2024).

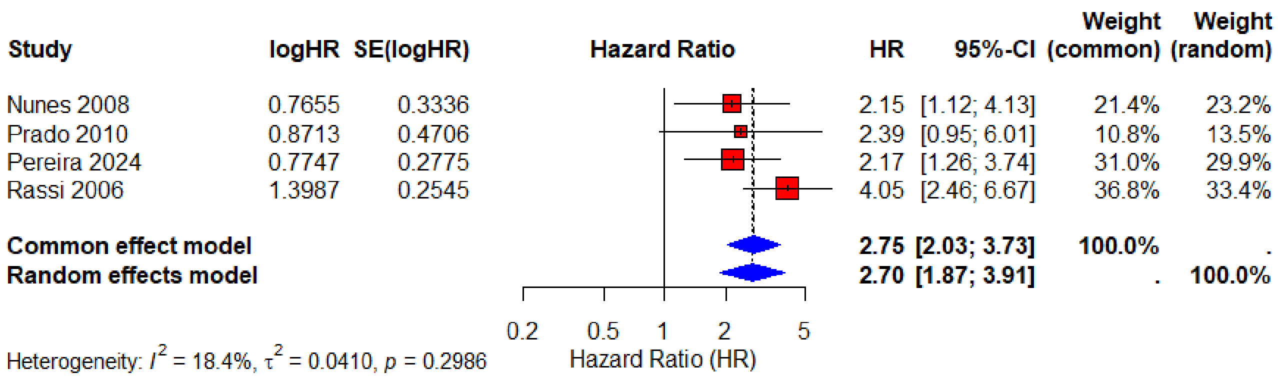

Supplement: Supplementary material Figure 1 [file 1678-9849-rsbmt-59-e0104-2026-md4.pdf]
