# Supplementary material for: Prognostic value of the New York Heart Association classification for cardiovascular events and mortality in Chagas cardiomyopathy: a systematic review and meta-analysis with GRADE recommendations
Source: Rev Soc Bras Med Trop. 2026 Aug 3;59:e0104-2026. doi: 10.1590/0037-8682-0104-2026 (PMC13432799; doi:10.1590/0037-8682-0104-2026)
Supplement: Supplementary material Figure 2 [file 1678-9849-rsbmt-59-e0104-2026-md5.pdf]

Supplementary Figure 2: GRADE assessment of evidence confidence.

Number of studies	Study design / Limitations	Risk of bias	Inconsistency	Indirectness	Imprecision	Publication bias	Other considerations	HR (95 % CI)	Overall Quality
**Mortality (NYHA III to IV vs I and II)**									
5	Cohorts	Not serious	Serious	Not serious	Not serious	Serious	None	HR* of 2.63 (95% CI: 2.00–3.45)	⊕⊕⊕◯ - Moderate
**Mortality (NYHA IV vs. I, II, and III)**									
2	Cohorts	Not serious	Not serious	Not serious	Serious	Serious	None	HR of 2.38 (95% CI: 1.36–4.15)	⊕⊕◯◯ - Low

*Evidence was downgraded for inconsistency due to heterogeneity in study populations and analytical approaches. However, no downgrading was applied for imprecision given the narrow confidence interval and the robust magnitude of the observed effect (HR >2).
